# Targeting SDF-1/CXCR4 to inhibit tumour vasculature for treatment of glioblastomas

**DOI:** 10.1038/bjc.2011.169

**Published:** 2011-05-17

**Authors:** D Tseng, D A Vasquez-Medrano, J M Brown

**Affiliations:** 1 Department of Radiation Oncology, Division of Radiation and Cancer Biology, Stanford University, 269 Campus Drive West, CCSR Room 1255, Stanford, CA94305, USA

**Keywords:** SDF-1, CXCR4, glioblastoma, vasculogenesis, irradiation, tumour regrowth

## Abstract

Local recurrence of glioblastomas is a major cause of patient mortality after definitive treatment. This review discusses the roles of the chemokine stromal cell-derived factor-1 and its receptor CXC chemokine receptor 4 (CXCR4) in affecting the sensitivity of glioblastomas to irradiation. Blocking these molecules prevents or delays tumour recurrence after irradiation by inhibiting the recruitment of CD11b+ monocytes/macrophages that participate in revascularising the tumour. We review the literature pertaining to the mechanism by which revascularisation occurs following tumour irradiation using experimental models. Areas of interest and debate in the literature include the process by which endothelial cells die after irradiation and the identity/origin of the cells that reconstitute the tumour blood vessels after injury. Understanding the processes that mediate tumour revascularisation will guide the improvement of clinical strategies for preventing recurrence of glioblastoma after irradiation.

Despite advances in techniques for administering radiotherapy, local recurrence of glioblastomas (GBMs) typically leads to patient mortality. In this review, we focus on the chemokine stromal cell-derived factor-1/CXC chemokine ligand 12 (SDF-1/CXCL12) and its receptor CXCR4 in affecting the sensitivity of GBM to irradiation. We begin with an overview of the discovery and characterisation of these molecules, followed by an examination of their role in tumour metastasis and tissue injury. Finally, we discuss the data and controversies surrounding their role in revascularisation and tumour recurrence. Our understanding of the role of SDF-1 and CXCR4 in tumour recurrence post irradiation is largely gained from pre-clinical studies using mouse xenografts of human tumours. The pre-clinical data will be the focus of this review. Clinical studies in this area are limited, but serve as an exciting area for future investigation.

The CXC chemokine family has multiple roles in leukocyte migration, immune response, angiogenesis, and tumour metastasis. CXC chemokines have four highly conserved cystine amino-acid residues, with a non-conserved amino acid separating the first two cystine residues. A second structural domain determines their function. The CXC chemokine family contains both angiogenesis-promoting and angiogenesis-inhibiting molecules. Stromal cell-derived factor-1 is regarded as an angiogenesis-promoting chemokine.

Stromal cell-derived factor-1 is a small pro-inflammatory chemoattractant cytokine that was first cloned by Tashiro *et al*, 1993, as a protein captured by its specific amino-terminal signal sequences. It was later identified functionally as a molecule involved in B-cell lymphopoiesis using an expression cloning strategy. Human CXCR4 was initially identified as a receptor for SDF-1 by screening chemokine receptor orphan genes for their ability to induce intracellular Ca^2+^ in response to human SDF-1. The mouse CXCR4 receptor was subsequently found by cloning candidate chemokine receptors and comparing the amino acid sequence with the human cDNA (Nagasawa *et al*, 1998). Recently, it has been shown that SDF-1 binds to another receptor, CXCR7 (Burns *et al*, 2006). This finding has opened up new possibilities for understanding the biology of SDF-1 and modulating SDF-1-mediated pathways.

Mice with genetic deletion of SDF-1 or CXCR4 are embryonic lethal and have defects in embryogenesis, haematopoiesis, cardiac ventricular septum formation, migration of neural precursors in the cerebellum, and blood vessel formation in the gastrointestinal tract (Nagasawa *et al*, 1998; Tachibana *et al*, 1998). The similar phenotypes of these mice suggest that CXCR4 may be the critical receptor through which SDF-1 acts during development. In the adult mouse, SDF-1 expression is found in lymphoid (bone marrow, thymus, and spleen) and non-lymphoid organs (brain, heart, lung, liver, kidney, stomach, and intestine). In the blood, CXCR4 expression has been described in peripheral blood monocytes, neutrophils, lymphocytes, and haematopoietic progenitors (Nagasawa *et al*, 1998), and has been shown to be involved in cell migration. Stromal cell-derived factor-1 has also been shown to induce migration of brain microglia and astrocytes (Tanabe *et al*, 1997). In the adult, SDF-1 functions as a chemoattractant for haematopoietic cells (Aiuti *et al*, 1997). In addition to its role in chemotaxis, the SDF-1/CXCR4 pathway has been reported to be a survival factor for human glioma cells through its upregulation of the PI3K/AKT pathway (Zhou *et al*, 2002).

CXC chemokine receptor 4 is a seven-transmembrane G-protein-coupled receptor that acts as a receptor for SDF-1 and is also a co-receptor for HIV entry into target cells. A discussion of CXCR4's role in HIV is beyond the scope of this review. We will instead briefly review what is known about signal transduction downstream of CXCR4 on binding SDF-1. Binding of SDF-1 to CXCR4 leads to phosphorylation of ERK2 and protein kinase B, coupled with activation of PI3-kinase and formation of PIP3 (Tilton *et al*, 2000). Stromal cell-derived factor-1-mediated signalling through CXCR4 has also been shown to mediate phosphorylation of focal adhesion proteins such as RAFTK, paxillin, and Crk, and transcriptional activation through p44/42 MAP kinase (Erk 1 and 2), and activation of NF-*κ*B (Ganju *et al*, 1998). As our current understanding of intracellular signalling pathways downstream of CXCR4 is largely through *in vitro* studies, it is not well understood to what extent these multiple signal transduction pathways are shared among the different cell types or between normal *vs* malignant cells.

## The SDF-1/CXCR4 pathway in tumour metastasis and tissue injury

The interaction between the CXCR4 receptor and its ligand, SDF-1*α*, has been shown to mediate tumour metastasis (Muller *et al*, 2001; Sun *et al*, 2005). Overexpression of CXCR4 has been detected in a variety of human malignancies, including breast, pancreatic, prostate cancer, and GBM. Muller *et al* (2001) demonstrated that CXCR4 is expressed in primary breast cancer cells and that SDF-1 was highly expressed in the most common sites of metastasis, including the lymph nodes, lungs, liver, and bone. When the CXCR4/SDF-1 interaction was blocked with a neutralising anti-CXCR4 antibody using an *in vivo* xenotransplant model, metastatic load was significantly reduced (Muller *et al*, 2001). These data support the role of the SDF-1/CXCR4 pathway in the formation of metastasis for some tumours.

The SDF-1/CXCR4 pathway is also involved in tissue repair (Kollet *et al*, 2003; Ratajczak *et al*, 2003). Kollet *et al* (2003) showed that CCl_4_-mediated liver injury led to an increase in the recruitment of human CD34^+^ progenitor cells by SDF-1 to the injured liver in NOD/SCID mice, suggesting that SDF-1 may direct haematopoietic progenitor cells to sites of tissue injury. However, whether these cells in the liver represent functional haematopoietic stem/progenitor cells (capable of reconstituting lethally irradiated hosts) was not examined. Chemotaxis of CXCR4-expressing murine muscle satellite cells towards SDF-1 has also been demonstrated, suggesting that migration of tissue-specific stem cells may be regulated by SDF-1 (Ratajczak *et al*, 2003).

## The role of SDF-1/CXCR4 in restoring functional vasculature in irradiated GBMs

Radiotherapy has a crucial role in the treatment of GBMs, but despite the very high radiation doses that can be delivered with new techniques such as high-dose boost stereotactic radiosurgery, the tumours invariably recur, leading to mortality in 75% of patients by 2 years after initial diagnosis. As most of the recurrences occur within the radiation field (Minniti *et al*, 2010; McDonald *et al*, 2010), any method of improving local control of the primary tumour by radiotherapy would improve the curability of patients with GBM. We have recently proposed that the cure rates for this malignancy might be improved by targeting the SDF-1/CXCR4 pathway, thereby preventing reconstitution of the tumour vasculature following irradiation.

In theory, reestablishing the tumour vasculature after radiation could be mediated either by sprouting angiogenesis (from adjacent blood vessels) or by circulating cells – including endothelial cells (ECs) or endothelial progenitor cells (EPCs), a process known as vasculogenesis (see the section ‘Restoration of tumour blood vessels after irradiation’). In pre-clinical models, several investigators have reported that radiation inhibits local angiogenesis, the generation of new vessels from surrounding vessels (Udagawa *et al*, 2007; Imaizumi *et al*, 2010). However, in these studies it is difficult to determine whether cells, locally or distantly, mediate tumour revascularisation after irradiation. To interrogate whether new vasculature formation relies on a different mechanism in the presence *vs* the absence of radiation, we examined the effect of radiation on the development of tumour vasculature in the absence of matrix metalloproteinase-9 (MMP-9), a key proangiogenic molecule in circulating CD11b+ cells. We demonstrated that tumours cannot grow in an irradiated site (given 20 Gy) of an MMP-9 knockout (KO) mouse but can grow in a non-irradiated MMP-9 KO mouse. Tumour growth is restored following irradiation if the bone marrow in the MMP-9 KO mouse is replaced with wild-type bone marrow (Ahn and Brown, 2008). Thus, MMP-9 from cells in the bone marrow transplant could restore tumour vasculature (determined by CD31 immunostaining and injection of Hoechst dye) and support tumour growth at a pre-irradiated site. This illustrated that revascularisation after irradiation required extracellular matrix modelling of MMP-9 by cells in the bone marrow, although tumour growth without irradiation did not, suggesting that they may depend on different pathways for recruiting new vasculature. We demonstrated through depletion experiments and immunostaining that CD11b+ cells mediate this effect. It is important to note that our finding that radiation prevents local angiogenesis is not the same as the proposal of Fuks and Kolesnick that radiation produces a rapid apoptosis of tumour ECs and vascular shutdown (Garcia-Barros *et al*, 2003). We and others who have measured tumour ECs after irradiation do not see this rapid apoptosis (Kioi *et al*, 2010; Kozin *et al*, 2010). Rather, we observe that ECs disappear slowly after irradiation over several days, consistent with a mitotically linked death.

CD11b+ monocytes/macrophages are important mediators of tumour revascuzlarisation and regrowth after irradiation. Neutralising the antibodies against CD11b+ monocytes/macrophages given after irradiation markedly enhances the antitumour effect of radiation in human tumour xenografted mice (Ahn *et al*, 2010). We observe that these monocytes also express CXCR4 on their surface. Inhibition of the interaction of SDF-1 and CXCR4 using the CXCR4 inhibitor, AMD3100, or antibodies against CXCR4 prevented the radiation-induced increase of CD11b+ monocytes/macrophages and inhibited revascularisation in intracranial human GBM xenografts. Moreover, these inhibitors abrogated or delayed the recurrence of the tumours following irradiation (Kioi *et al*, 2010). Potentiation of the anticancer drug BCNU by AMD3100 has also been shown with the intracranially implanted U87 human glioblastoma, though the mechanism for this may have involved abrogation of the survival function of the SDF-1/CXCR4 pathway in the tumour cells (Redjal *et al*, 2006) rather than prevention of revascularisation after irradiation.

**Figure 1:**
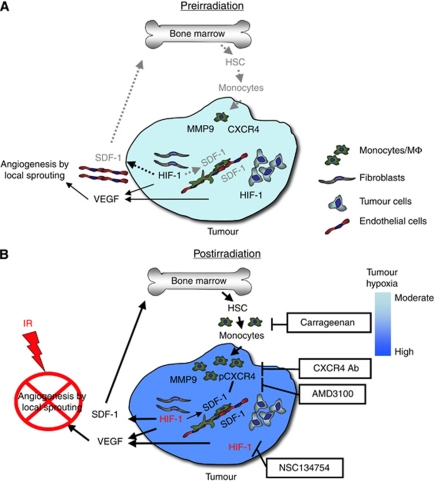
Model of the main contributions of bone marrow-derived cells (BMDCs) and cytokines that promote restoration of tumour vasculature following irradiation. Prior to irradiation, tumour growth is governed largely by local angiogenesis. When local angiogenesis is inhibited by irradiation, growth of the tumour vasculature (essential for recurrence of the tumour) can only occur from circulating cells, of which BMDCs are an essential component. Following irradiation, the tumour becomes more hypoxic and HIF-1 is increased as the tumour attempts to regrow. This induces SDF-1 and promotes the recruitment of CD11b+ monocytes/macrophages and retention of these cells in the tumour. Stromal cell-derived factor-1/CXC chemokine receptor 4 is the key interaction for the influx of BMDCs as AMD3100, an inhibitor of CXCR4/SDF-1, and antibodies against CXCR4 block the recruitment and/or tumour retention of the BMDCs, inhibit restoration of the tumour vasculature, and prevent tumour recurrence. The various inhibitors and the points in the cycle at which they act are shown in boxes. Reproduced from Kioi *et al* (2010) with permission. (**A**) Pre-irradiation; (**B**) post-irradiation.

We hypothesise that elevated levels of tumour SDF-1 caused by increased tumour hypoxia (resulting from gradual loss of ECs) following irradiation lead to accumulation of CXCR4-expressing monocytes/macrophages in the irradiated tumour. This is also suggested by data in the literature showing that the recruitment and retention of proangiogenic hematopoietic cells to sites of ischaemic tissue damage or to tumours is mediated by the interaction of the SDF-1 with CXCR4 (Ceradini *et al*, 2004; Aghi *et al*, 2006; Jin *et al*, 2006). Stromal cell-derived factor-1 functions as a hypoxia-inducible gene through the action of the transcription factor hypoxia inducible factor-1 (HIF-1). We have shown that irradiated tumours gradually lose vasculature after irradiation, thereby becoming increasingly hypoxic and upregulating HIF-1. Consistent with this, the HIF-1 inhibitor NSC 134754 prevents both the radiation-induced tumour accumulation of CD11b+ monocytes/macrophages and prevents tumour recurrence (Kioi *et al*, 2010). We and others have shown that SDF-1 levels are increased in irradiated tumours (Kioi *et al*, 2010; Kozin *et al*, 2010) and in the plasma following local tumour irradiation of intracranial GBM (unpublished data). A model showing the pathway by which CD11b+ monocytes/macrophages accumulate in irradiated tumours is shown in Figure 1. Also shown in Figure 1 are the points in the pathway that have been targeted therapeutically to prevent the regrowth of irradiated tumours in pre-clinical studies.

## Restoration of tumour blood vessels after irradiation

Vasculogenesis is a term used in embryology to denote the *de novo* formation of blood vessels. Its use in the present context would imply that all the cellular components of the tumour vasculature after irradiation come from circulating cells, not from residual vascular cells in the tumour that survive radiation, nor from surrounding angiogenic vessels. We hypothesise that ECs do not regrow from surviving ECs in the radiation field at the doses used in our studies (15–20 Gy) or at TCD50 doses (doses that control 50% of the tumours) typical for transplanted tumours (40–100 Gy), and particularly in SCID mice in which all the stromal cells are highly radiosensitive (Budach *et al*, 1993).

Experimental data show the importance of bone marrow-derived CD11b+ monocytes/macrophages for the restoration of tumour blood vessels after irradiation. Unlike CD11b+ monocytes/macrophages in non-irradiated tumours, these cells express the angiopoietin-2 receptor Tie-2 as well as MMP-9 and the F4/80 macrophage marker, and are therefore classified as Tie-2-expressing macrophages (TEMs). CD11b+ cells from an irradiated tumour share many of the same properties as ECs and have been shown to interact with newly forming tumour blood vessels (De Palma *et al*, 2005). Whether or not there exists EPCs in the BM that directly give rise to tumour endothelium has been a source of controversy and confusion in the literature. To address whether cells in the bone marrow directly give rise to the components of blood vessels, we carefully examined the ECs in irradiated tumours in mice with Lac-Z or GFP-expressing bone marrow. From such studies, we and others concluded that the ECs in the tumour do *not* arise from cells in the bone marrow (Ahn and Brown, 2008; Kioi *et al*, 2010; Kozin *et al*, 2010). To our knowledge, the so-called bone marrow-derived ‘endothelial progenitor cells’ have not been shown directly to form tumour vasculature. For instance, Purhonen *et al* (2008) have shown using a parabiotic mouse system (two mice joined so as to have a common blood supply) that VEGFR-2+ bone marrow cells did not incorporate into the tumour endothelium. Other investigators have shown using either orthotopic aortic allografting (Hillebrands *et al*, 2002) or a parabiotic mouse system combined with reverse bone marrow transplantation (Aicher *et al*, 2007) that non-bone marrow-derived circulating ECs or EPCs from the liver and small intestine home to sites of active angiogenesis. Thus, circulating ECs, which are not derived from cells of the adult bone marrow, may be recruited to the irradiated tumour. We hypothesise therefore that circulating ECs or EPCs migrate to the irradiated tumour, thereby reconstituting the vasculature. Such a possibility would fit the existing data but needs further experimental studies to validate the hypothesis. The stimulus for this colonisation by ECs is likely to be SDF-1 via its receptor CXCR4 (Orimo *et al*, 2005) and/or its receptor CXCR7, which is highly expressed in tumour-associated blood vessels including those in GBM (Miao *et al*, 2007; Liu *et al*, 2010). CXCR7 has also recently been shown to be co-expressed with CXCR4 on cortical interneurons and to be essential for their SDF-1-stimulated migration (Sanchez-Alcaniz *et al*, 2011). CD11b+ cells in the irradiated tumour may also help recruit ECs into the tumour or help reorganise the tumour extracellular matrix via MMP-9 to mediate revascularisation. Thus, the restoration of the tumour vasculature following radiation doses similar to those used in cancer treatment could formally fit the definition of vasculogenesis (i.e., derived entirely from circulating cells), but further work is required to prove this conclusively. Other groups have explored alternative hypotheses by which tumours establish and/or maintain their vasculature, including endothelial differentiation of GBM stem-like cells (Ricci-Vitiani *et al*, 2010). However, markers used to define ECs can be expressed by normal stem cells and cancer stem cells, and it does not necessarily indicate that GBM stem cells possess plasticity towards an EC fate. A subset of GBM stem cells may acquire features shared by ECs when grown under EC-promoting conditions *in vitro*, although this may not reflect their behaviour *in vivo*. Our lab has been focusing on the mechanism of revascularisation in the post-irradiated GBM tumour, as this can be interrupted to dramatically improve the outcome in pre-clinical studies. We are actively interested in identifying the source of ECs in the post-irradiated GBM tumour.

## Clinical data

Our data suggest that the pathway of post-irradiation recruitment of CD11b+ monocytes/macrophages in tumours may be important not only in mice but also in human patients. We have shown by immunohistochemistry that the levels of CD11b+ monocytes/macrophages are higher in GBM recurrences than in the same tumours prior to therapy (Kioi *et al*, 2010). Whether or not these cells mediate revascularisation of human GBM after irradiation is not yet known. However, understanding the pathway involved in local recurrence of GBM after radiotherapy holds much clinical potential for preventing recurrence of GBM.

Recently, a clinical study of 95 patients with GBM reported recurrence in 79 patients (83%) after a median follow-up period of more than 18 months. Although the majority of recurrences were located in the radiation field, in 17 patients GBM recurred outside the radiation field. As recurrences outside the radiation field were observed after a longer follow-up time, different mechanisms may be responsible for in-field and out-of-field recurrences (Minniti *et al*, 2010). Blocking tumour revascularisation after irradiation by inhibiting vasculogenesis would be the most relevant method to prevent recurrences of GBM in the radiation field and this strategy would benefit the majority of patients with GBM. However, if out-of-field recurrences became the prominent pattern of recurrence after local tumour irradiation, whole-brain irradiation could be given, as this has been a viable clinical option for GBM in the past. Improved markers or imaging for identifying patients at risk for in-field *vs* out-of-field recurrence would allow us to select patients who would benefit from targeted radiation *vs* whole-brain irradiation. Preventing revascularisation of tumours after irradiation would be important for both radiation treatment strategies.

## Conclusions

Stromal cell-derived factor-1 is a small pro-inflammatory chemoattractant cytokine that binds to its G-protein-coupled receptor CXCR4. The interaction of SDF-1 with CXCR4 has been shown to play a role in tumour metastasis by CXCR4-expressing tumour cells migrating to normal tissues expressing SDF-1. In tissue remodelling after injury, haematopoietic cells migrate to sites of ischaemic injury, where increased levels of SDF-1 are produced by the hypoxic upregulation of HIF-1. It has recently become apparent that migration and recruitment of circulating proangiogenic monocytes/macrophages can occur in tumours following local irradiation. We have proposed that the increased hypoxia seen in tumours following irradiation recruits CD11b+ monocytes/macrophages and ECs to the tumour, thereby restoring the tumour vasculature. The reliance of the tumour on revascularisation after irradiation suggests a promising therapeutic approach involving inhibition of this pathway. There are potentially a number of ways to achieve this, including inhibition of HIF-1, antibodies against CD11b or against CXCR4, and pharmacological inhibition of the SDF-1/CXCR4 pathway with the drug AMD3100 (plerixafor). These strategies have been successfully used in pre-clinical studies. Of these, the use of plerixafor is currently the most promising for further investigations in human clinical trials as a proof of principle, as this drug is already approved for clinical use. Understanding the mechanism by which tumours revascularise after irradiation offers promising therapeutic strategies for treating GBMs.
